# Musculoskeletal rehabilitation in controlled trials: Is it correct to compare different types of exercise?

**DOI:** 10.1590/1516-3180.2024.0374.29012025

**Published:** 2025-05-19

**Authors:** André Pontes-Silva

**Affiliations:** I Postgraduate Program in Physical Therapy, Department of Physical Therapy, Universidade Federal de São Carlos (UFSCar), São Carlos (SP), Brazil.

**Keywords:** Osteoarthritis, Musculoskeletal system, Pain, Exercise, Fibromyalgia, Low back pain, Clinical trial [publication type], Strength Training, Time-Under-Tension, Spine, Musculoskeletal Disorders, Physical Activity, Physical Exercise

## Abstract

**BACKGROUND:**

There are several randomized controlled trials (RCTs) in the literature on musculoskeletal rehabilitation that compare different types of exercise; however, the comparison is not relevant because the groups generally perform different physical efforts, and the researchers are not aware of this, nor do they control for the confounding variables.

**OBJECTIVES:**

To discuss the methods of comparison of different types of exercises in musculoskeletal rehabilitation.

**DESIGN AND SETTINGS:**

Short communication developed at the Universidade Federal de São Carlos (UFSCar), São Carlos (SP), Brazil.

**METHODS:**

A narrative review of the motion cadence, time-under-tension, actual duration of an exercise session, and total physical effort was conducted.

**RESULTS:**

To compare the different types of exercise, it is crucial that the parameters of the proposed exercises are the same between the groups, i.e., the exercise intensity, total physical effort, and actual duration of the exercise session.

**CONCLUSION:**

It is correct to compare different types of exercise, however, in the field of musculoskeletal rehabilitation, RCTs adequately controlled for these variables are lacking.

## INTRODUCTION

Exercise repetitions are a widely used parameter in randomized controlled trials (RCTs) of musculoskeletal rehabilitation. However, research results can be manipulated because the number of repetitions does not account for the motion cadence,^1^ time-under-tension,^2^ actual duration of the exercise session,^3^ and total physical effort,^4^ which are the variables with the most significant impact on the outcomes.^5^

### Motion cadence: a repetition divided into four phases

**Figure 1:**
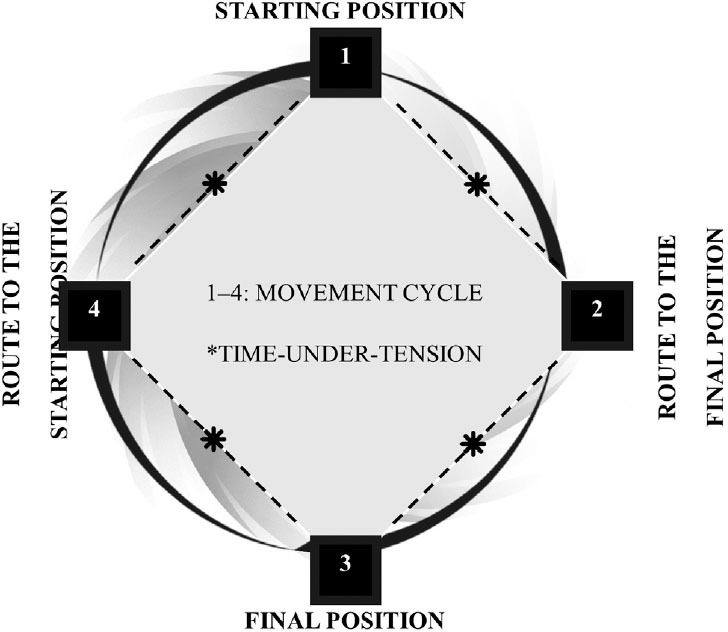
Movement cycle showing the four phases of a movement and time-under-tension.

When muscles contract to overcome resistance during physical exercise, the complete movement of the muscles and joints involves the following four phases (Figure 1): (1) starting position; (2) movement to the final position; (3) final position; and (4) movement back to the starting position.^2^

*Time-under-tension (seconds): time of physical effort expended in the repetition. 1–4 (movement cycle): 1=starting position, 2=route to the final position, 3=final position, 4=route to the starting position.

Notably, in each of these four phases, a physical effort (energy expenditure) is needed^1,6^ either to maintain a position against the resistance of the exercise (Phases 1 and 3; e.g., an isometric contraction) or to move through a range of motion (Phases 2 and 4; e.g., an isotonic contraction). The sum of these four phases characterizes the time-under-tension.^7^

### Time-under-tension: the sum of the four phases

Time-under-tension refers to the total duration for which a muscle or group of muscles withstands mechanical stress during exercise. The duration of a movement (cadence) impacts time-under-tension and muscle activity, which ultimately affects the outcome being researched.^2,7^ It is possible to maintain the same number of repetitions while manipulating the time-under-tension.^8^ This may be observed in the two examples described below.

Example 1: A patient performing 10 repetitions with 60 seconds of time-under-tension takes an average of 6 seconds for each movement (6 × 10 = 60) and 1.5 seconds for each of the four phases of the movement (1.5 × 4 = 6). Example 2: A patient performing 10 repetitions with 90 seconds of time-under-tension takes an average of 9 seconds for each movement (9 × 10 = 90) and 2.25 seconds for each of the four phases of the movement (2.25 × 4 = 9) (Table 1). Thus, time-under-tension is the variable that may determine the actual duration of an exercise session.

**Table 1:** Representative example of someone manipulating time-under-tension while maintaining the same number of repetitions

Time-under-tension (seconds)	Repetitions (number)	Movement cycle (Phases 1-4; Figure 1)	Cadence (seconds)	Total time movement cycle (seconds)
10	10	4	0.25	1
20	10	4	0.50	2
30	10	4	0.75	3
40	10	4	1	4
50	10	4	1.25	5
60	10	4	1.50	6
70	10	4	1.75	7
80	10	4	2	8
90	10	4	2.25	9
100	10	4	2.50	10
120	10	4	3	12
140	10	4	3.50	14
160	10	4	4	16
180	10	4	4.50	18
200	10	4	5	20

### Actual duration of an exercise session

The time-under-tension may increase or decrease without altering the number of repetitions. This implies that patients in the experimental group (e.g., those undergoing resistance exercise) may exert less (or more) physical effort than those in the control group (e.g., those undergoing aerobic exercises). The level of effort required is dependent on the therapist’s intention during the intervention.^9^

To show the total/actual duration of an exercise session, it is necessary to sum and describe (in minutes or seconds) the time-under-tension of all exercise sets and subtract the time used for rest intervals.^9^ Furthermore, the actual duration of the exercise session may not be sufficient for a fair comparison, as it is necessary to evaluate the total physical effort to ensure that the intensity and volume of the exercises are equivalent between the groups.^10,11^

### Total physical effort

When comparing different exercise programs, such as resistance exercises versus aerobic exercises or Pilates versus strength training, it is important to ensure that the programs being tested are at the same intensity level, e.g., moderate versus moderate, and provide the same total physical effort for the actual duration of the exercise session.

Different exercises performed at the same intensity may have different total physical effort.^4^ Since it is known that the amount of physical effort, rather than the type of exercise, is the component that affects clinical outcomes, it is important to check units of total physical effort to ensure that there are no differences between the groups.^4^

## CONCLUSION

To compare different types of exercise, it is crucial that the parameters of the proposed exercises are the same between the groups, i.e., exercise intensity, total physical effort, and actual duration of the exercise session. As such, it is correct to compare different types of exercise, however, in the field of musculoskeletal rehabilitation, RCTs adequately controlled for these variables are lacking.
